# Online cultural heritage as a social machine: a socio-technical approach to digital infrastructure and ecosystems

**DOI:** 10.1007/s42803-025-00097-6

**Published:** 2025-03-12

**Authors:** Javier Pereda, Pip Willcox, Gustavo Candela, Alexander Sanchez, Patricia A. Murrieta-Flores

**Affiliations:** 1 Towards a National Collection, Edinburgh, UK; 2 Liverpool John Moores University, Liverpool, UK; 3 Oxford, UK; 4 Universidad De Alicante, Alicante, Spain; 5 Lancaster University, Lancaster, UK

**Keywords:** Phrases: Digital Infrastructures, Cultural Heritage, Digital Collections, Decolonial Digital Infrastructures, GLAM, CCS CONCEPTS, Digital libraries and archives, Information Integration, Cultural characteristics

## Abstract

The advent of digital technologies has profoundly transformed cultural and heritage sectors, providing new avenues for broader access and interactions with digital collections. This shift has enabled Online Cultural Heritage (OCH) to evolve into an extensive ecosystem. Given the complexity that emerges from these networks and stakeholders, it is crucial to develop a clearer understanding of the extensive terminology used in the sector and establish pathways to deconstruct this complexity. Therefore, this article's aim is threefold: 1) it examines how OCH ecosystems foster the ongoing reinterpretation and recontextualisation of cultural heritage collections through technologic innovations and the Web. In doing so, it highlights the relevance of policy development and the establishment of ethical frameworks that address both human and technical complexities of Cultural Heritage (CH) knowledge; 2) using the Open Archival Information System (OAIS) as a framework and its terminology, the article maps the workflows and socio-technical actors of the OCH ecosystem; and 3) the article applies Callon’s Process of Translation, a methodology for understanding how socio-technical networks evolve and use it to critically deconstruct digital infrastructures in OCH. This methodology enables the contextualisation and reinterpretation of cultural narratives across digital platforms, both online and offline, underscoring the dynamic interplay between technology, human agency, and cultural context. We explore how OCH ecosystems and other infrastructural ecosystems aid in preserving and facilitating engagement with open knowledge and research, and function as complex networks of cultural institutions interconnected through knowledge infrastructures. Whilst the paper places the primary approach within UK infrastructures, it provides alternative perspectives from the Global South, particularly Latin America, to contrast and further illustrate a reflection on the current and future challenges behind a sustainable OCH ecosystem, its implications for further networks, and its potential as a model beyond the CH sector. Furthermore, this framework can become paramount to identifying obstacles and opportunities for digital infrastructures, establishing a nuanced understanding of OCH as a core infrastructural element in the generation of knowledge from digital collections or digital infrastructures around the world. Finally, we provide a glossary of terms to establish a common ground between the wide range of parties involved in OCH. CCS CONCEPTS • Digital libraries and archives • Information Integration • Cultural characteristics.

## Introduction

Digital technologies have transformed the cultural and heritage sector, offering unprecedented access to cultural collections. This shift has enabled Online Cultural Heritage (OCH) to evlolve into a dynamic ecosystem where digital collections serve as live spaces for cultural exchange and knowledge representation. The OCH ecosystem and infrastructures can be conceptualised as a ‘social-machine’, where human agency and technology interact to manage and disseminate digital collections. To analyse these socio-technical interactions, this paper applies Callon’s (1984) **‘Process of Translation’**, to explore how actors form alliances and networks through diverse negotiation stages, ultimately shaping the sustainability of a digital ecosystem. In addition, the Open Archives Information System (OAIS) framework (CCSDS, 2012; Lavoie, 2000) is used to contextualise concepts, terminology, actors and network interactions.

While this study focuses on UK-based infrastructures, it incorporates decolonial perspectives to contextualise these socio-technical systems more broadly. Previous studies highlight the infrastructural role of digital cultural heritage (Ross et al., 2024), where OCH, along other infrasructures, supports digitisation, preservation and reuse of both tangible and born-digital heritage objects. However, OCH ecosystems extends to netwoked systems including *knowledge infrastructures* and governance. This article applies the **Process of Translation** to analyse network interactions through -problematisation, interessement, enrolment and mobilisation-, revealing how OCH ecosystems negotiate control, access and representaiton. Furthermore, in the following Sect. (2) we establish a shared understanding of the technical terminology to ensure clarity and accessibility for a diverse readership by reviewing the literature on digital transformations, and present OCH as a socio-technical system where technology and social structures shape access to Cultural Heritage (CH) collections. Section 3 aligns the OAIS framework with the **Process of Translation** to contextualise how socio-technical processes take place within the digital preservation of the CH ecosystems. The findings present case studies that emphasise the critical importance of inclusivity, representation, and ethical considerations in digital practices. Finally, in Sect. 4, we implement the **Process of Translation** to help understand the broader implications of the digital transformations of the OCH ecosystem and the CH sector.

## The digital transformation of cultural heritage and socio-technical systems

Cultural Heritage (CH) includes tangible and intangible elements, such as artefacts, traditions, and knowledge inherited from previous generations (UNESCO, 2022). It encompasses regional and national identities, oral history and music, and community characteristics (Cleere, 1984; Plets, 2016). Museums, libraries, and galleries have traditionally preserved and managed these materials, each using specialised methods. However, they are now adapting to a broader and connected digital landscape (Candela et al., 2020; Padilla, Scates Kettler, & Shorish, 2023a; Padilla, Scates Kettler, Varner, Shorish, & 2023b). This revolutionary shift, often termed the ‘digital turn’ (Nicholson, 2013), involves navigating the socio-political context and acknowledging the dynamic social construction of digital platforms and cultural heritage. Such transformation can also pose challenges to the community through commodification and data extraction, loss of world-systems and cultural impositions, and even the loss of local knowledge (Pinto, 2018). That said, the impact of digital colonialism is not necessarily a direct result of the digital spaces. As part of the CH and OCH ecosystems, digital spaces exist wihtin a broader network of infrastructures, including data infrastructures (e-infrastructure),^1^ knowledge-access systems (e.g. scolarly social machines), and legal and policy frameworks that govern access and preservation (Pinto, 2018).

The Semantic Web has been pivotal in this digital transformation, providing frameworks for interoperability by integrating and contextualising information from diverse sources (Berners-Lee, 2006). Its primary function is to connect data, enabling complex queries and deeper insights. Linked Data, a core Semantic Web technology, helps structure, define, and link data, allowing CH organisations to integrate datasets across and beyond their institutions (Candela, 2023, 2024; Gracy, 2015). As a result, traditional methods for describing and cataloguing collections has changed (Corcho & Hyvönen, 2024; Hyvönen, 2025). In their place, Web technologies, aggregators and search engines now serve as primary access points, reshaping the narratives once controlled by physical institutions. Museums, for instance must now address the multidimensional nature of both tangible and intangible items in their collections (ICOM, 2007, 2022). Similarly, libraries, tasked with the role of preserving books and documents, use both physical and digital pathways for engagement, such as exhibits and consultation (Diez, 2013:35). Historically, archives have consisted of predefined collections of records from a particular organisation, collected based on their original use within an institution (Bettivia, 2016). However, digital archives transcend the physical record, developing unique characteristics within computing environments (Duranti & Thibodeau, 2006). In digital spaces, CH narratives converge, merging different ways of describing and presenting cultural material, expanding, challenging or providing alternative displays of knowledge beyond where objects are held, described or stored.

Supporting OCH requires diverse communities, disciplines, ecosystems and infrastructures. In the UK, this includes government investments in CH preservation through film, television and creative industries, such as UKRI’s Creative Industries Clusters (DCMS, 2023) and CoSTAR (UKRI, 2024a), which specialises in gaming, TV and digital entertainment. The Creative Industries and CH sectors, both integral to OCH, require interactive tools to engage with digital content. Additionally, high-performance computing supports Digital Humanities and Heritage Science, backed by AHRC initiatives such as Research Infrastructure for Conservation and Heritage Science (RICHeS) (UKRI, 2024b) and the Distributed System of Scientific Collections UK (DiSSCo) (DiSSCo, U. K 2024). Lastly, animal, botanic, and zoo knowledge intersect with cultural heritage, particularly in curated displays, linking OCH to tourism as another intersectoral domain.

**Fig. 1:**
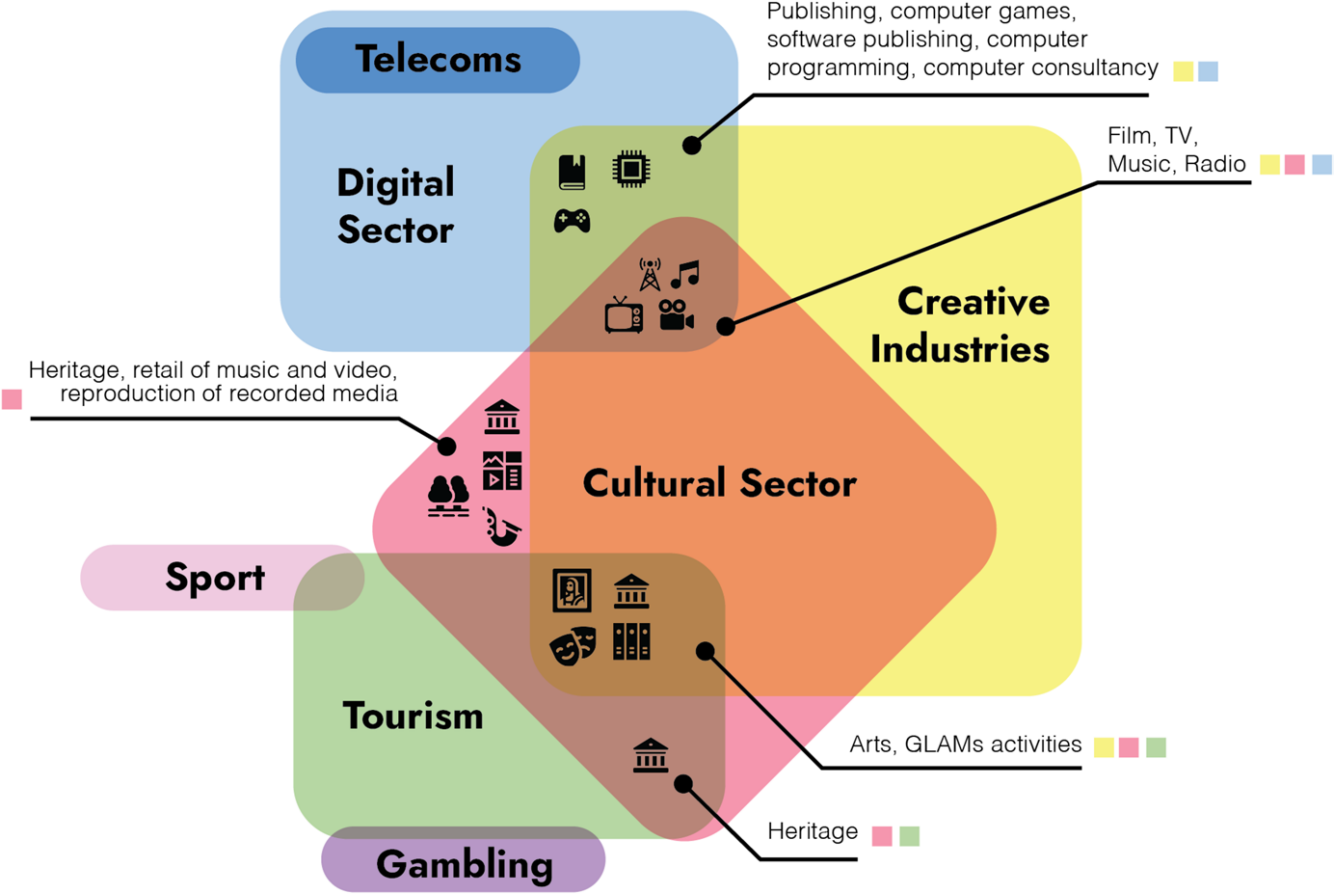
The intersection of the Cultural Sector and SIC areas adapted from DCMS (DCMS, 2022)

To help map and contextualise this complexity, we used the Standard Industrial Classification (SIC) (Fig. 1) to classify and map the impact of the cultural and heritage sector across diverse domains and align them to economic areas. This stratification helps frame the sector’s broad socio-technial relationships. In this paper, CH is understood as the intersection of economic areas (SIC), and knowledge and heritage that transcend disciplinary boundaries, especially when placed on the Web. While the ‘cultural sector’ and ‘cultural heritage sector’ have distinct meanings, both involve preserving, managing, and promoting tangible and intangible cultural assets. They also manage, produce, and promote cultural activities and engagement, which are not limited to either sector. SIC mapping illustrates their economic, social, and technical networks, including: 1) the Digital Sector through interface design, visualisation, and analysis; 2) the cultural sector through digitisation, arts, public programs, and educational content; and 3) tourism and museums through exhibits, city guides, and websites promoting travel.

### Building a socio-technical definition of online cultural heritage

Digital transformations or innovations can be categorised as *disruptive* or *sustaining* (Hughes & Cosier, 2001; Kostoff et al., 2004). *Disruptive innovation* introduce ground-breaking technologies, that reshape organisational paradigms and create new products or services. For instance, CH organisations use conceptual reference frameworks and ontological models to describe collections. They have also expanded access to broader audiences and social groups while transforming how information and knowledge are produced and engaged, particularly through Application Programming Interfaces (API). In contrast, *sustaining innovations* enhance existing services, improving accessibility and performance to meet specific user needs. The Web, for example, has facilitated democratic knowledge engagement and enabled diverse communities to participate in research and cultural knowledge production. In many cases, this includes communities that had been neglected and isolated from their own cultural knowledge.

Understanding knowledge production and sharing in the OCH ecosystem and CH sector clarifies the roles and behaviours of actors within the network (Doerr, 2009). Despite more people are using computers to analyse digital collections, many lack the documentation explaining the affordances and meaning behind the knowledge they contain. Datasheets provide a standardised approach to documenting OCH and CH data and processes (Alkemade et al., 2023). Aided with an understanding of digital transformations, this can help reveal how socio-technical power structures are built in the OCH ecosystem. Semantic Web technologies have created a cross-domain-knowledge-space that integrate conceptual and organisational frameworks across CH institutions, including Galleries, Libraries, Archives, Museums (GLAM), universities, archaeological sites, and community groups. Consequently, CH organisations must establish dataset policies that can -this is referred to as the *application profile* or *metadata crosswalk* (DCMI, 2024; Gaitanou et al., 2012). This is to say that CH organisations must integrate organisational and epistemological perspectives to create shared understandings and agreements (Allemang & Hendler, 2011; Antoniou & Van Harmelen, 2008), which shape the understanding of collections, objects and knowledge about them. Objects, metadata and digital surrogates no longer exist in isolation, but must be contextualised to meet the needs of sectors and disciplines within CH sectors and SIC areas. According to the Open Archive Information System (OAIS), this integration of object and process forms an *information object,* which represents information and metadata aligned to both organisational and user needs. In OCH, they are essential for creating sustainable platforms that provide relevant data and services (Lavoie, 2000). To ensure that, *information objects* embody ‘meaningful’ information to the communities and their technologies, the concept of ‘Collections as Data’ can be applied (Thomas Padilla et al., 2023a, 2023b). This framework offers an approach to manage and use collections in alignment with the FAIR^2^ principles ensuring transparency, reusability and reproducibility including algorithms, tools, workflows, pipelines, paradata, software, scientific publications, and exhibitions used to generate new knowledge (Lamprecht et al., 2020), and safeguarded by CARE^3^ principles across different technological platforms.

#### The OAIS model as a framework to link information objects and humans in OCH

The OAIS framework defines six core compliance elements: 1) Negotiate for and accept appropriate information from information producers; 2) Obtain sufficient control of the information provided to the level needed to ensure Long-Term Preservation; 3) Determine, either by itself or in conjunction with other parties, which communities should become the *designated community* and, therefore, should be able to understand the information provided; 4) Ensure that the information to be preserved is independently understandable to the *designated community;* 5) Follow documented policies and procedures which ensure that the information is preserved against all reasonable contingencies, and which enable the information to be disseminated as authenticated copies of the original, or as traceable to the original; and 6) make the preserved information available to the *designated community* (CCSDS, 2012).

Both OAIS and FAIR consider the role of humans and information objects within a single socio-technical space, though their connections remain underdeveloped. For example, if the information is ‘independently understandable to the *designated community*’, it implies that the communities (or the *information objects*) should have the ability to convey ‘meaning’ without the help of specialists or experts. Thus, the relationship between *designated communities* and *information objects* is also paramount to define the sustainability of an information system, especially if they are aligned with the OAIS model and aim to engage with FAIR principles.

*Designated communities* are the potential users, linked to the ‘meaningfulness of *information objects* through their knowledge and representation. However, when representation is absent -as with many subaltern groups and data from the Global South- how can these *designated communities* expected to understand *information objects* within an OAIS model? Similarly, how can software, algorithms, and other *information objects* engage with communities outside hegemonic institutions such as corporations (economic infrastructure), government (surveillance), and research universities (science), which handle *information objects* at scale? (D'ignazio and Klein, 2023). While OAIS offers no guidance on defining *designated communities*, it is suggested that they are shaped through institutional missions and mandates (Kärberg, 2014). However, relying on institutional frameworks risks reinforcing exclusion, as structuring resources around predefined *designated communities* may marginalise groups whose knowledge systems or access methods fall outside standard formats (Bettivia, 2016). Aligning *designated communities* with FAIR and CARE principles helps define how they engage with data and which tools, licenses, and policies are ‘meaningful’ to them. Although FAIR principles promote uniform technological and knowledge access, acknowledging the diverse -information and knowledge- needs of *designated communities* is essential to expanding ethical frameworks into data justice, and knowledge equity (D'ignazio and Klein, 2023).

Both *sustaining* and *disruptive* technologies shape our understanding of CH and how to engage with it. Technology has significantly increased the complexity around *information objects* and broadened their use across diverse *designated communities.* It has also enabled SIC sectors to contribute while facilitating knowledge production and access. However, gaps remain in the understanding of how technologies and social interactions that emerged from the creation of the Web have influenced and continue to influence the various areas (or networks) that benefit from, sustain, or provide an infrastructure to produce, manage and engage with CH content (*information objects*). To navigate this complexity, we conceptualise the CH sector (and the Cultural Sector) as a social-machine, focusing on interaction between *information objects* and *designated communities* on the Web. Specifically, we apply Callon’s **Process of Translation** (Callon, 1984) as a socio-technical methodology to deconstruct, contextualise and clarify the networks operating within and beyond the CH sector.

#### Contextualising the social and technical aspects of online cultural heritage

OCH as a social-machine emerges from the intersection of technological and human systems within a socio-technical ecosystem. This ecosystem fosters dynamic interactions among cultural institutions, technologies and diverse (*designated*) communities, reshaping access, engagement, and the interpretation of cultural heritage collections. To analyse these interactions, we use the **Process of Translation** which consists of several key stages: **problematisation, interessement, enrolment, mobilisation,** and **stabilisation**. These stages form the core framework for mapping the complex web of relationships that define the OCH ecosystem. Introducing these concepts early establishes a foundation to deconstruct the CH sector and the OCH ecosystem. We aim to provide a foundational understanding of how digital tools and platforms are integral to the negotiation of cultural narratives in the OCH ecosystem. We will make use of the OAIS digital terminology as a workflow aligned to the SIC sector and further social and human enterprise.

*Information objects* support dynamic interactions and relationships, such as those found in annotated datasets, bots, and algorithms. Their relevance to *designated communities* depends on specific information needs, which activate the **Process of Translation**, enabling network interactions. These interactions foster collaboration and integrate members, creating self-governing, self-functioning social-machines. A holistic approach to deconstruct the OCH ecosystem moves beyond individual, secular, or organisational perspectives, reducing traditional boundaries. For instance, libraries may initiate the archival or curatorial process of an object (e.g. an illustration) using controlled vocabularies or conceptual reference models (CRM) like Dublin Core to suit their workflows. Meanwhile, museums may describe the same item from a different conceptual perspective, using a different CRM. This is the case for illustrations by Leonardo Da Vinci held at Spain’s National Library and the Museo Nacional del Prado. Although both institutions describe similar or identical objects, they use distinct cataloguing processes. One aspect that illustrates the role of CH organisations on a broader network, is that the way an *information object* is introduced into the ecosystem defines the role of the CH organisations in the network and not necessarily how a particular technology might be used. While CRMs can impose a specific *techno-economic paradigm*, communities from other networks can still re-use and repurpose the information and contribute back with information as user or community generated -digital- content. In some cases, these community networks overshadow the original institutions, as most OCH searches occur via Google Scholar or Wikipedia (Blackwood, 2014; European Commission, 2013; Guldbæk Rasmussen et al., 2009). Some libraries have even considered integrating ‘bootleg’ content into their systems (Harrop et al., 2015). As a result, CH organisations are gradually adapting to socio-technical changes, embedding themselves into the OCH ecosystem (Marty, 2009; Marty, 2014; Trant, 2009).

OCH as a social-machine exists within broader networks or social-machines that generate, share and apply knowledge (meaning), known as *Knowledge Infrastructures* (Edwards et al., 2013). *Knowledge Infrastructures* exist in the **Process of Translation** through scholarly social-machines, which focus on the inclusive and collaborative knowledge generation ecosystem, and thus help overcome customary barriers to scholarly activity (De Roure & Willcox, 2020). The OCH shares similarities with Web-based social-machines, particularly in human–computer collaboration, social system functionality and bio-technologic dependency, where the system ceases to exist without human or technical enggement. However, OCH is not strictly a Web-based system. While it leverages Web-based tools, some contributions remain within internal intranet-servers, forming local clusters that interact with *Knowledge Infrastructures*, elements of Social Computing, including Digital Cultural Heritage infrastructures. Although *information objects* and their knowledge may not be part of the Web, there are great benefits from these being on it, particularly from the Digital Humanities perspective. They can also offer access to knowledge to other social and scholarly networks beyond OCH (Toscano, 2021). In addition, there might be scenarios where CH organisations might not be on the Web, but still contribute to the OCH ecosystem through the sharing of data (*information objects*) or the development of Web tools or policies (Pereda, 2016). Understanding how CH organisations and *designated communities* engage, share, and interact with the OCH ecosystem is therefore essential.

#### Diversifying what can be online cultural heritage

To differentiate OCH from terms such as virtual museum, digital museum, museum on the Web, digital archive, or any specific ‘digital’ or ‘virtual’ element, it is essential to view OCH as a hybrid space functioning both online and offline. This perspective highlights challenges related to the Web, including decolonisation and the influence of AI, the Semantic Web and Web-based technologies. Insights from Critical Heritage have showcased the role of constructivist approaches, which help provide agency to present-day audiences in the production of knowledge, as well as integrate heritage as an integral part of social and cultural movements. Most importantly, it serves as a necessary first step in critiquing and self-critiquing Western heritage practices -not only to do it better but to do it critically (Kirshenblatt-Gimblett, 2023). In addition, Critical Heritage begins to align with some elements around Indigenous governance and CARE principles, contributing to the understanding and developing creative solutions to social, economic and ecological problems which arise as a result of conflicts between different -world- systems and different cultural groups. It advocates for the participation of local communities and helps provide a strong ethical focus on narrative control. With regard to OCH, Critical Heritage envisions ‘digital heritage’ as a process to be deconstructed rather than a mere technical implementation for preservation, management, accessibility and governance (Harrison et al., 2023)*.* Critiques have highlighted how Web information systems often exclude indigenous groups by imposing internet-centric norms, like Open Access, which pressures CH groups and *designated communities* to follow approaches that conflict with their practices (Ogden et al., 2015). However, it is crucial to deconstruct both social and technical elements of *Knowledge Systems*, not just the Web. While indigenous groups may be absent from socio-technical infrastructures, their exclusion stems from colonial systems within *Knowledge Infrastructures*. Open Access as a colonial imposition is part of a ‘state domination’, where the Global North Imposes its values on the Global South (Fontana, 2002). Such perspective imposes hegemonic impositions of how it should be implemented, influencing legislation, regulation and institutional practices (Albarez Gómez, 2016). Open science and access are outcomes of historical shifts tied to the Global North’s construction of knowledge (García Guerrero, 2024). Bourdieu’s (1988) ‘Homo Academicus’ explains how social hierarchies in academic spaces shape the *Knowledge Infrastructures*, often excluding indigenous groups. In a Western context, this exclusion leads to the ‘subalternisation’ of people, reducing them into historical objects rather than subjects of knowledge, thereby marginalising their lived experience (Alpízar, 2017). In this case, Critical Heritage still fails to specify workflows for authority control, self-determination, and knowledge production within Western *knowledge systems*. OCH extends beyond just CH systems and encompasses broader spaces and *knowledge infrastructures.* As we present in the examples below, many of the approaches in Critical Heritage around authority control, self-determination and how these deploy within applicable frameworks have been concepts long implemented outside Europe and especially outside Anglo-Western spaces. In addition, whilst a ‘critical turn’ in the engagement with heritage and media studies has been taken place, there is still a lack of scrutiny yet to be equally carried out on the digital transformation of the infrastructures. Organisations like Association for Computer and the Humanities, the Alliance of Digital Humanities Organisations (ADHO) focus predominantly on the Global North, marginalising the Global South (Ibekwe, 2022: 75). This marginalisation stems not from a lack of data, but from a failure to engage with the *knowledge systems* of the Global South. For example, the National Institute of Anthropology and History and the National Autonomous University of Mexico actively integrate indigenous participation in their *knowledge infrastructures* (INAH, 2019; Tlachia, 2012; UNAM, 2012). Alternatively, most Web-based *knowledge infrastructures*, developed in the Global North reflect a Western worldview and are often ill-equipped to manage this type of knowledge (D'ignazio and Klein, 2023; Ibekwe, 2022). For indigenous communities, implementations of ‘open access’ in digital archives from predominantly white institutions are marked by extraction, cultural appropriation and theft (Carbajal & Caswell, 2021). Therefore, it is argued that Western spaces, and thus Critical Heritage, remain limited in the deployment of CARE principles and Indigenous data sovereignty and governance due to the lack of diversity imposed through monocultural pipelines that privilege a select group of creators, subjects, languages and perspectives (Carbajal & Caswell, 2021). Considering CARE principles, it is important to explore how knowledge is produced, and introduced to platforms, and transformed to align with the worldview and world-system of the *knowledge infrastructures* from which it originates before being deployed within a digital infrastructure. In response, Global South initiatives, including Latin America and Africa, have redefined Open Access and Open Science from their own worldview or cosmovision. Their approach focused on strengthening knowledge production (*infrastructures*) as an open knowledge economy for the benefit of the wider society (Vila Viñas & Barandiaran, 2015). Examples such as ‘*El Buen Vivir’* (Good Living) integrate indigenous wellbeing perspectives into open science, extending it into ‘*El Buen Conocer*’ (The Good Knowledge) to challenge the influence of capitalism on knowledge (cognitive capitalism) and dominant socio-technical systems (Vila Viñas & Barandiaran, 2015). Decisions around digital records are direct manifestations of the diverse layers of values and social responsibility, and thus, their infrastructures are a reflection of that (Carbajal & Caswell, 2021), which include the technology, academic practices and digital practices.

Despite these challenges, it is important to acknowledge that within an OCH ecosystem, there is no ‘single Web’, or unified *knowledge infrastructure*. It should be noted that terminology such as Open Access, Open Science, and Open Data shouldn’t be restricted to a specific worldview. For example, Mexican indigenous groups use of their colonial and historical archives for legal defence and land restitution. Indigenous historical documents such as the seventeenth-century *títulos primordiales* are sets of land deeds based on pre-hispanic forms of organisation that can include paintings and maps that are seen as proof of indigenous presence and occupation of land and ownership since pre-colonial times. The *títulos primordiales* were used after the revolution in 1920 by Emiliano Zapata through the *Plan de Ayala* for land restitution in Ixmaquilpa, and that same legislation was then used in 1976 and in 1982. Indigenous groups and the government established the Agrarian Code in 1940 that explicitly granted indigenous land ownership through the Recognition and Entitlement of Communal Property in the Mexican Constitution under Article 27 (Ruiz Medrano, 2010:215). This law article was later amended in 1991 by the Mexican president Carlos Salinas de Gortari after the implementation of the NAFTA treaty, which prompted a wide range of neoliberal policies and the exploitation of indigenous groups and their natural resources. Most importantly, this legal reform changed the perception (cosmovision) of these documents by non-indigenous groups into historical, anecdotal excerpts. The current legal interpretation of such documents relies merely on the political inclination of individual judges and their perception of such documents. However, recent examples are still emerging. This is the case of the settlement of 40,076 hectares in Santiago Niltepec in Oaxaca, where in 2006, the community used the *títulos primordiales* from 1713, and a current ongoing legal dispute in San Miguel Chignautla, Puebla (Ruiz Medrano, 2010). For this reason, opening access to indigenous *knowledge infrastructures,* which might include complex datasets such as the *títulos primordiales,* could help setting a legal and cultural precedent for policy and legislation changes, recognition and validation, legal advocacy, cultural revitalisation, and public awareness and education. However, open access policies must be aligned with CARE principles and initiatives launched from ‘*Buen Conocer’*, such as the FLOK^4^ society. It also needs to be considered that opening data and knowledge to the community will depend on the readiness of CH organisations and *designated communities,* including their *knowledge infrastructures*, have to intake and make sense of the *information objects*. Critical Archives and Critical Heritage should incorporate indigenous data sovereignty initiatives and place authority and control in the hands of the community that represents the data subjects, as well as provide clear instructions for non-indigenous outsiders on how best to proceed with this data (Carbajal & Caswell, 2021). Finally, it is important to develop both the human (social, political) and technology frameworks to understand the ways in which CH organisations and other social-machines in OCH as *designated communities* might establish collaborative knowledge sharing and production through *information objects*, which include economic models, tools and software, as well as digitisation and digitalisation initiatives.

### Encapsulating a definition of OCH as a social-machine

The Web and, and by extension OCH ecosystem, operates as a system where people (communities/organisations/society) act alongside automated services provided by machines and computers (technology) (N. R. Shadbolt et al., 2013). These socio-technical relationships or dependencies, are known as “social-machines”. Social-machines are typically co-managed by developers and end-users (Berners-Lee & Fischetti, 2000; Hendler et al., 2008; Shadbolt et al., 2019; N. R. Shadbolt et al., 2013), as in the case with OCH. Moreover, social-machines function as ecosystems supporting the Web’s infrastructure, highlighting the OCH ecosystem’s role in connecting to other social-machines.

To understand the OCH as a social-machine, three aspects are critical (De Roure et al., 2015; Halford et al., 2010).

First, technology and society are co-constituted -digital platforms and tools in the CH sector are shaped by their users (*designated community*). In turn, these technologies influence how the sector is perceived and how *designated communities* engage with digital collections.

Second, OCH operates as a networked ecosystem sustained by collaboration between digital technologies and human agents who preserve, manage, and engage with cultural heritage. All actors -whether human or technical- play an equal role in how cultural items are understood and presented on digital platforms, extending beyond the Web.

Lastly, performance is a crucial factor. The Web is constantly evolving, and this dynamic transformation is mirrored in the OCH ecosystem. It constantly transforms how knowledge production networks (or social-machines) interact with cultural heritage collections, whether through digitisation, datafication, digitalisation, curation, archival processes, or sharing content through APIs. In this evolving ecosystem, knowledge is structured around *information objects*, which may still be tied to physical surrogates or locations like GLAM institutions. However, *information objects* are not passive entities -they actively shape the interaction between *knowledge infrastructures* and *designated communities*, continuously driving the evolution of the ecosystem (Waller, 2017).

The role of OCH as a social-machine has created deeply intertwined relationships between users, technology, and the knowledge surrounding cultural heritage collections, where one cannot exist without the other. This interdependency between people, organisations and technology is referred to as the *techno economic paradigm* (Williams & Edge, 1996). A key example is the widespread adoption of Content Management Systems (CMS) and Digital Asset Management Systems (DAMS), which have become standard in the sector. While most organisations rely on these systems, the *technologic trajectory -*the pathway and rate of technological evolution- has outpaced many institutions, creating significant challenges in adapting to digital transformation. As a result, job roles in the sector often fail to reflect the technical expertise now required, particularly in areas related to the Web and digital content (Dziekan, 2014), and data science (Boon et al., 2025). In the UK, most CH organisations remain dependent on legacy systems, limiting their ability to fully integrate into the OCH ecosystem despite their willingness to participate (Gosling et al., 2022). Simultaneously, the *techno economic paradigm* within CH organisations and the broader OCH ecosystem has disrupted traditional hierarchical structures, transforming them into more complex, decentralised networks where information flows in multiple directions (Hughes & Cosier, 2001). Due to the novelty and recent adoption of these technologies, new organisational models are emerging, necessitating a fresh approach to building resilient, sustainable infrastructures for both cultural and SIC sectors. This shift also demands innovative policies and strategies, with the OCH ecosystem at its core.

## The process of translation as a method to deconstruct the och ecosystem

Having considered the complexity of socio-technical systems within the OCH ecosystem, this section introduces the **Process of Translation** as a framework for identifying the interactions among actors within the OCH network. This methodology examines technologic adoption and organisational challenges in the OCH ecosystem, focusing on integrating further members and producing a strategic understanding of technology and social groups, while reducing constructivist and technologic determinisms (Cordella & Shaikh, 2003).

This section will finalise the integration of the social and organisations workflows that take place in the *metadata crosswalk*, contextualising them through the OAIS reference model. It builds upon the definition of OCH and applies the **Process of Translation** to explore how social norms and power structures, such as metadata standards and technology-based economic models, influence the development and adoption of technologies.

One of the main advantages of using Callon’s **Process of Translation** is its ability to highlight the role of different stakeholders in shaping the direction and outcomes of technological advancements within the *techno economic paradigm* and the *metadata crosswalk* (Callon, 1984). Given these factors, the **Process of Translation** will involve five core stages: [1] **Problematisation,** [2] **Interessement,** [3] **Enrolment,** [4] **Mobilisation,** and [5] **Stability or** **Finalisation.**

### Problematisation

The **Problematisation** phase begins when disruptive or innovative technology is introduced to a network. In this case, the network is the OCH ecosystem. Actors start identifying and articulating the problem that the innovation aims to address. The OCH ecosystem relies on data that generates information and, subsequently, knowledge. By providing data that is meaningful to end users, data is transformed into information and further into knowledge (Ackoff, 1989). This describes how *information objects* are produced. Within the OAIS reference model, *data objects,* knowledge-bases, and actors of the OAIS environment (production/ingest, management and engagement/consumption) merge with presentation and rendering formats, producing *information objects (*Lavoie, 2000*).*

There are two important aspects of *information objects.* First, they allow both social and technical actors of the OCH ecosystem can generate knowledge. Second, they guide how *designated communities* (as in the OAIS environment) or potential users engage with the data in meaningful ways. This engagement occurs through a range of tools, software, data models, communication, and organisational approaches. Academics, scholars, curators, and community members act as knowledge producers, with *information objects* as central actors in the network. Knowledge production -through curation, research, cataloguing, data visualisation tools, annotation systems and publications- forms a common process in the OCH ecosystem. These actors can belong to various social organisations in the OCH ecosystem and the CH sector, as well as SIC areas, including independent research organisations (IRO) such as GLAMs, software companies, or independent researchers.

This network structure and its social-technical relationships produce what is known as the **Obligatory Passage Point (OPP)** (Callon, 1984). The **OPP** occurs when different actors -both technical and human- must pass through a specific node to achieve the goals set in this **problematisation** phase. Applying the **Process of Translation** to the **OPP** clarifies the power dynamics and influences within the OCH ecosystem. This approach reveals how specific scientific and *technical innovations* shape and influence research and knowledge production in the cultural heritage sector.

In the OCH ecosystem, it is crucial to recognise the network dynamics and the potential OPP, whether social or technical. Technology has certainly eased the interoperability, collaboration, and access to knowledge, especially for CH organisations. They have adopted technological developments such as the Semantic Web, Web technologies such as APIs, and Open Access, Open Standards, and Open-Source initiatives. These advances have created both opportunities and challenges, such as those involving copyright, access, licensing, and decolonisation of conceptual representations. Socio-technical changes have also led to *sustained innovation* by improving the quality of *information objects* that describe items in collections.

Beyond CH organisations, groups from other fields -such as Computer Scientists, Human Computer Interaction scholars and those in SIC areas, including Creative Industries- engage with *information objects,* often through APIs and Open Standard Technologies. For instance, the European Union, has focused on creating of digital collaboration platforms for cultural institutions, such as the European Collaborative Cloud for Cultural Heritage (European Commission et al., 2022) and the European Data Spaces (European Commission, 2025). Their report identifies common issues such as digitisation, legal concerns, and resource limitations (*techno-economic paradigm*), and highlights how these challenges can unite actors through **interessement**. Given the complexity of the ecosystem, the **Process of Translation** offers a valuable framework for examining the relationships of actors. It also helps identify information needs of *designated communities*, including those in SIC areas, and frame them with specific *techno economic paradigms,* and transactional relationships with *information objects*.

As part of this evolving landscape, decentralised identifiers (DID) provide a way to verify digital assets without relying on central authorities. Unlike traditional identifiers such as URIs, DIDs support self-description, verification and interaction through authentication methods and service endpoints, aligning with FAIR principles, enabling secure access, audit trails, and compliance with ethical and legal governance frameworks. When integrated with Smart Contracts, DIDs can enhance digital preservation, ensuring transparency, provenance tracking and controlled reuse of digital assets (Bashir, 2020; Preukschat & Reed, 2021), which is especially relevant in the alignment of indigenous data governance, CARE principles through data spaces and platforms’ managing cultural collections.

Throughout the **problematisation** phase, actors strive to reduce barriers to publishing collections as data for small and medium organisations and align to common goals that foster a sustainable or stable ecosystem. For example, academic publishing and museography networks benefit from engaging with *information objects*, and reusing them for their own needs (Meier zu Verl & Horstmann, 2011). However, for this cycle to take place, knowledge production/engagement remains essential (Fyson et al., 2013). That said, data spaces should help facilitate data sovereignty and reduce the creation of data silos whilst promoting data economy, and providing reproducible methods and workflows, both on the data as well as the technologies used to engage with the data, as in the case of Jupyter Notebooks (Candela et al., 2023). The next section will explore how actors form **systems of alliances** based on socio-technical common goals, which ultimately help stabilise the network (Callon, 1984).

### Interessement

When actors in the network align to solve challenges presented by the innovations in the *techno economic paradigm,* they form clusters based on their interests or information needs identified during the **problematisation** phase. This begins producing power dynamics within the network, driven by perceived benefits (*sustainable innovations*) or challenges (*disruptive innovation*). This marks the second phase of the **Process of Translation** called the **interessement** phase.

During **interessement**, the network mobilises support by strengthening relationships between socio-technical actors and creating **systems of alliances**, also referred to as the **triangle of interessement**. An example is the rapid development of research in the humanities, linguistics, and social sciences, driven by methods from Computer Science, Machine Learning, Software Design, and Digital Humanities. These interdisciplinary approaches have transformed information management processes within both physical CH organisations, and digital actors from the OCH ecosystem. However, while the **triangle of interessement** or **system of alliances** strengthens specific relationships, it can also weaken others. Analysing these triangulations highlights techno-centric impositions around Open Access and knowledge representation systems imposed by the Global North onto indigenous groups and the Global South. This issue can persist despite the **systems of alliances** formed between academic institutions, IROs and SIC areas, which tend to exclude actors from other Knowledge Infrastructures such as cultural practitioners, non-profits and local or indigenous communities. Processing centres address these imbalances by providing a shared infrastructure for large-scale data management, reproducibility and equitable access (Candela et al., 2024), fostering collaboration and inclusion across diverse networks.

Many actors OCH ecosystem may have weaker connections due to factors such as geography, language, economy, or digital capacity. By deconstructing socio-technical infrastructures, stakeholders can identify the specific *techno economic paradigm* of their network and establish more sustainable **system of alliances.**

Partnerships and negotiations must consider challenges affecting sustainability within the *techno economic paradigm*. Actors such as organisations establish *technologic trajectories* such as data management plans, contribution agreements, governance and knowledge representation systems. Weak ties in the OCH ecosystem often stems an inability to recognise the relevance of tools, software, and their relationship with *information objects.* Additional factors include a lack of expertise in integrating community-based practices and knowledge infrastructures from *designated communities,* as well as limited digital capacity and capability in the CH sector (Taylor et al., 2022). The **Process of Translation** helps identify how **multilateral negotiations** must occur to generate **interessement**, leading to **enrolment** and creating sustainable **systems of alliances** (Callon, 1984).

### Enrolment

**Fig. 2:**
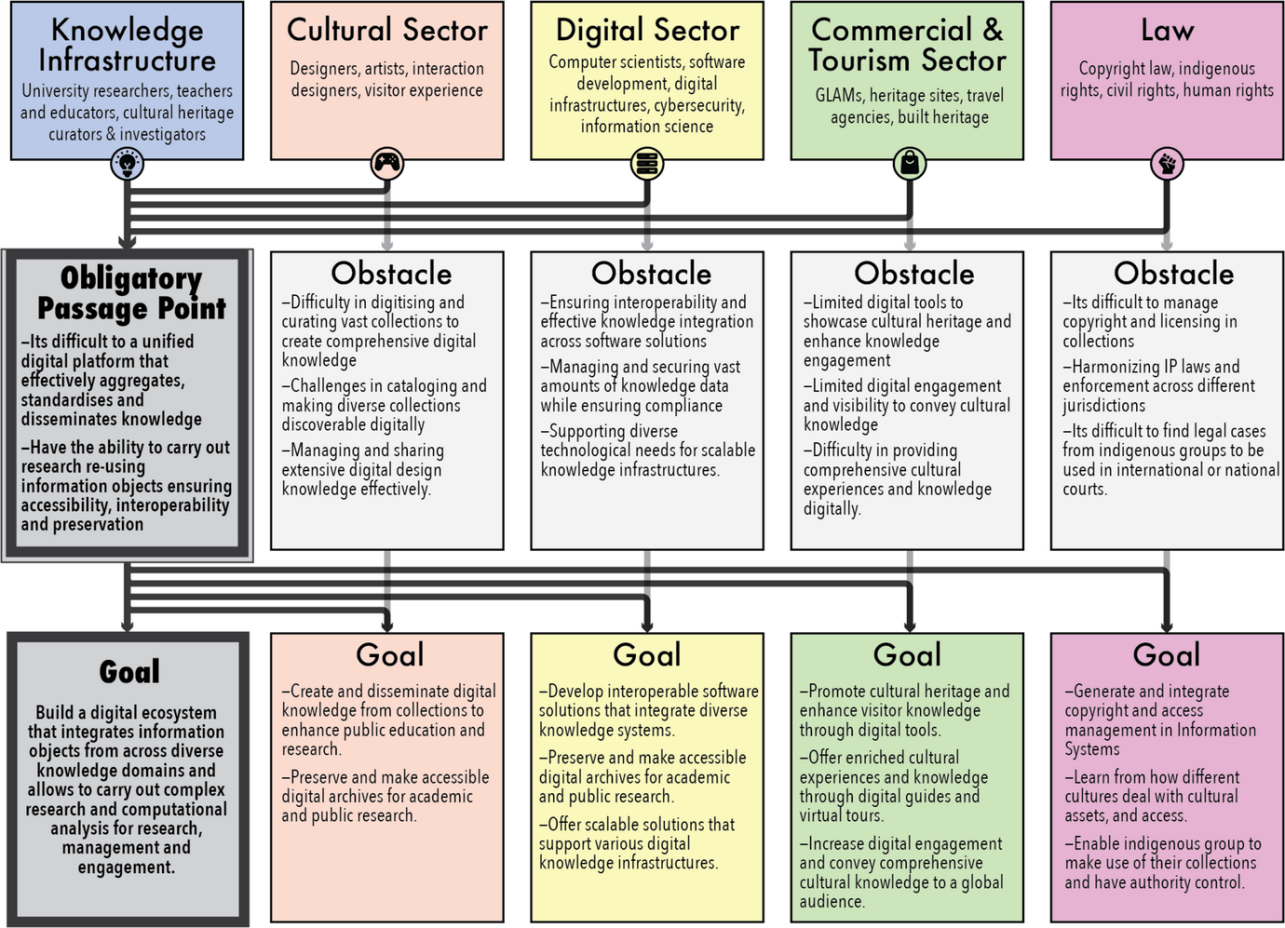
This illustration presents an example of some of the different sectors that can be involved in Online Cultural Heritage. While each can have multiple individual goals and confront their own problems, they will all converge at an **Obligatory Passage Point** if they aim to build a digital ecosystem that integrates information objects across knowledge domains. The OCH **Obligatory Passage Point** emerges from the need of access to information to produce engagement tools for knowledge

For innovation to take place, the diverse networks or social groups, such as, research communities, research councils, and policymakers must engage in the process of **enrolment**. Through this process, negotiations between the actors will begin establishing responsibilities for the network members (multilateral negotiations). The planning of negotiations should make the most of the contributions from the actors into the development of knowledge production through the *information objects*. Several **systems of alliances,** particularly from the computer sciences, digital humanities and technology SIC areas have already established strong **triangles of interessement**, where it is important to continue nourishing such network connections. For example, research councils in the UK have initiated **enrolment** by investing in computational infrastructures in the Humanities. The UK Research and Innovation (UKRI) initiatives of infrastructural fund to engage with cultural and heritage networks such as Research Infrastructure for Conservation and Heritage Science (RICHeS), Convergent screen technologies and performance in real-time (CoSTAR), the Distributed System of Scientific Collections (DiSSCo) and Towards a National Collection (TaNC),^5^ are key examples of the exploration and establishment of infrastructural ecosystems to establish and initiate **multilateral negotiations** between social actors and the technologies needed to support them. Other initiatives can also engage with other **multilateral negotiations** that can help establishing further **systems of alliances**. While on the one hand UKRI’s World Class Labs (WCL)^6^ funding such as Capability for Collections Fund (CapCo),^7^ and Creative Research Capability (CResCa),^8^ aims to build the core physical set of technologies including facilities and equipment, alongside the skills required to operate and maintain them. On the other hand, the case of government implementations such as UK Innovation Strategy,^9^ Levelling Up the United Kingdom,^10^ and the UK Digital Strategy.^11^ In the case of the European Union and the European Collaborative Cloud for Cultural Heritage (ECCCH), they have aimed to provide a system of ambassadors adopted by several organisations and cultural institutions. Their vision is that by integrating these groups of actors into strategic governance of the cloud, it will help identify areas for improving relationships between small and large organisations and promote interactions that are not typically part of the daily activities of those actors within that part of the ecosystem (European Commission et al., 2022) (Figs. 1 and 2).

**Fig. 3:**
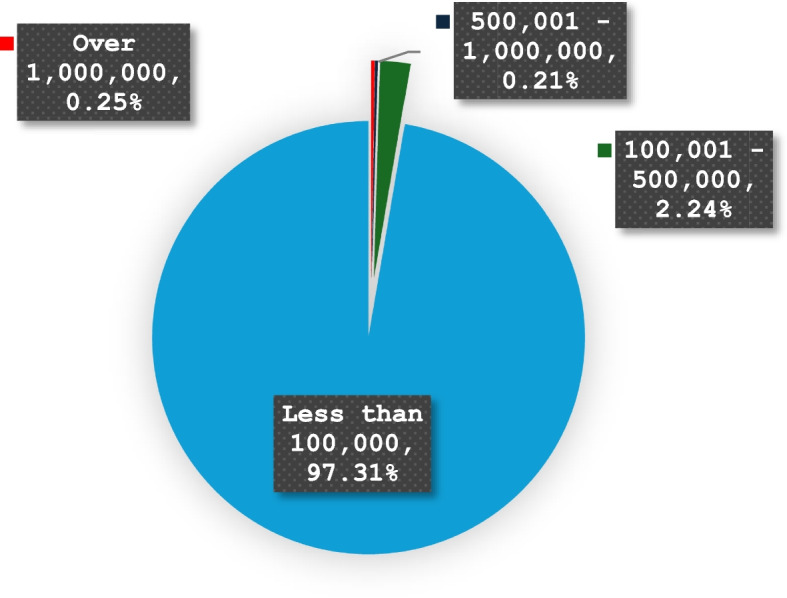
Percentage of cultural heritage organisations sharing content through Europeana (as in 14 February 2025)

When actors engage in **multilateral negotiations** that generate **enrolment** through **interessement**, it is relevant to acknowledge that not all actors will have the same disposition to become part of the network. It is also essential to consider the limitations of knowledge, economic, geographic, and cultural infrastructures in enabling sustainable **multilateral negotiations.** In the case of the OCH ecosystem, **enrolment** can become challenging due to factors such as empowerment through representation, legal and ethical compliance, cultural and infrastructural inequities, trust, governance, and cultural illiteracy. As a result, these factors can produce barriers that limit the ability of actors to join the networks. This is known as the **resistance** phase. On the one hand, in the OCH context, CH organisations often struggle to access the wide range of *technologic innovations* from the ecosystem. For example, in the UK it is estimated that only about six organisations hold more than 60% of the country’s cultural data and assets (Gosling et al., 2022). Similarly, in large collaborative initiatives such as Europeana, it can be seen that over 90% of organisations share less than 100,000 items, and less than 0.25% of organisation share over a million records (Fig. 3). On the other hand, technologic developments such as AI and big data methodologies can sometimes lead to unintended consequences, such as misrepresentation of subaltern knowledge. If information and *information objects* are not meaningful for the actors/communities (*designated communities*), including subaltern -*designated communities*, there is a risk of colonial impositions through external systems from centric institutions such as corporations (economic infrastructure), government (surveillance and monitoring) and research universities (science), which have the capacity to work with *information objects* in a large scale (D'ignazio and Klein, 2023). In this context, actors are required to explore the potential hegemonic results that the generation of diverse **triangles of interessement** can have on other subaltern groups. The development of **interessement** and **enrolment** requires actors to foster trust, ensure cultural sensitivity, facilitate shared decision-making and governance, comply with legal and ethical standards, and empower of communities through representation. Furthermore, actors cannot assume that all participants will be able or are willing to take part in their **enrolment** to the ecosystem or investment pipelines, as ignoring the rights to pre-informed consent and voluntary consent can lead to human rights abuses against subaltern groups, with indigenous groups being the most affected (Gallegos Bolaños & Flores Geldres, 2023; Palmater, 2021).

### Mobilisation

**Fig. 4:**
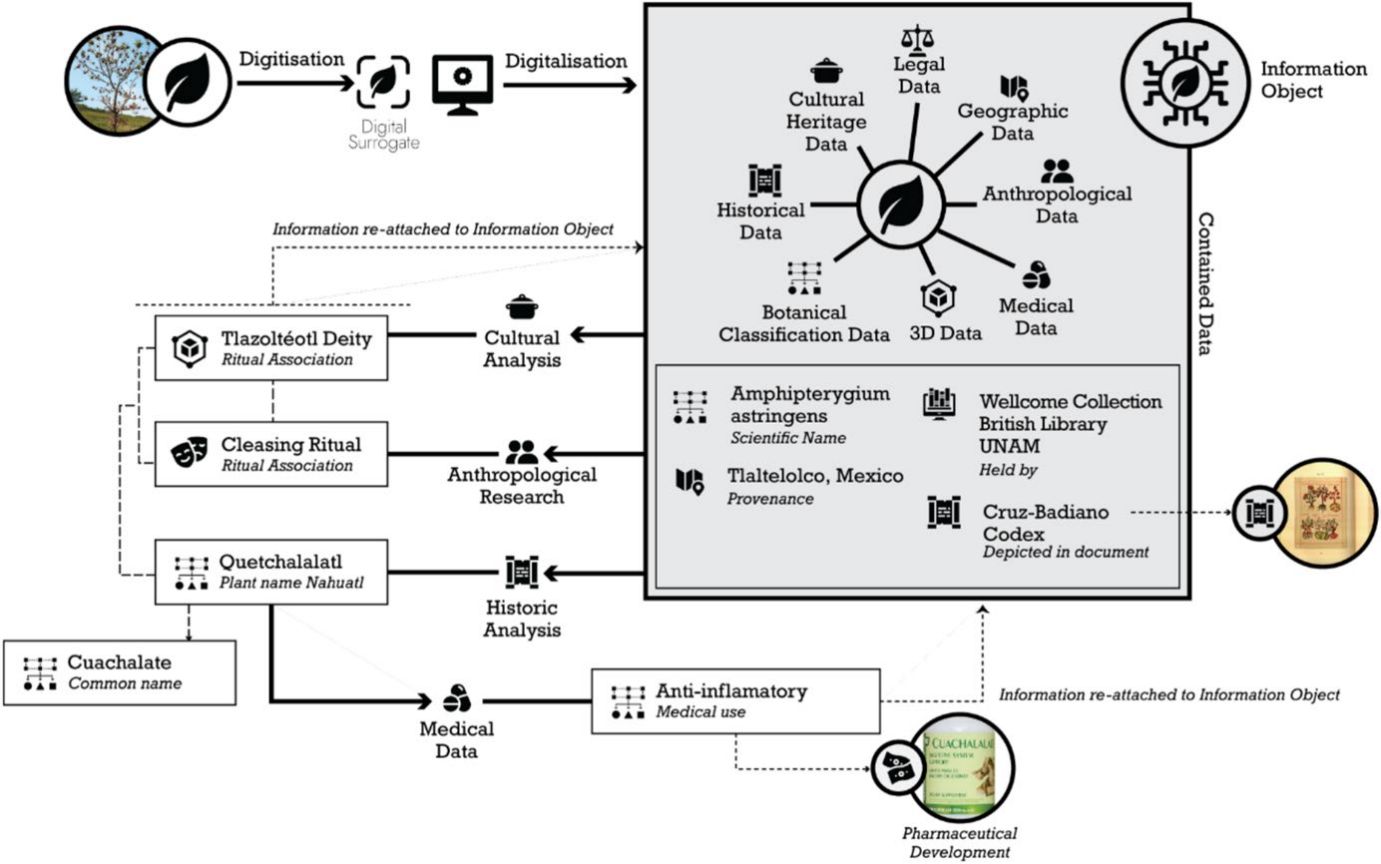
Example of knowledge exchanges with the data from an information object In this example, the information object is an image of a endemic American plant commonly known as *Cuachalalate*. The original Nahuatl (Aztec) name is *Quetchalalatl*, it was associated to the goddess *Tlazolteotl*, and used in cleansing rituals. It continues to be used as a medicine and in medical research. It can be found in historical sources, scientific databases, and anthropological research, among other sources

Once the network has identified the potential benefits and drawbacks of putting *innovation(s)* in place, negotiations between actors need to take place. This initiates the **mobilisation** phase where *innovation* will be adapted and refined in response to feedback. The main objective is to align all actors for the **stability** or **finalisation** of the network. For example, in the UK, the **system of alliances** between academics, academic institutions, and the government has assisted generating initiatives such as the Arts and Humanities Data Service (AHDS) (Burnard & Short, 1994), and the Archaeology Data Service (ADS) (Richards, 1997). The councils and researchers identified the need to develop an infrastructure to collect and preserve digital collections, for it to be re-used in research and teaching at universities (Beagrie, 1999). The AHDS considered the different levels of **resistance** such as the geographic and disciplinary distribution of the network as part of their **enrolment.** They distributed the service having an Executive base at King’s College London, and service providers such as ADS at York University, AHDS at Essex University, the Oxford Text Archive at Oxford University Computing Centre, the VADS at Surrey Institute of Art and Design and PADS at Glasgow University (Beagrie, 1999; Burnard & Short, 1994). This level of **enrolment** and **mobilisation** has helped the network gain stabilisation/finalisation, and the ADS has been able to initiate further **enrolment** with actors from other disciplines and beyond the UK (Fig. 4).

For the development of the OCH ecosystem, it is important that positive **mobilisation** takes place. This calls for a strategy that makes use of inclusivity policies, ethical practices such as the CARE principles, and cultural sensitivity. This development requires a range of stakeholders and cultural representatives to address different needs and perspectives, thus reducing data and information biases. Furthermore, the engagement with communities and obtaining consent for the use (and sharing) of their cultural heritage is another key element of this development. Infrastructural technologies must be both economically accessible and sustainable, whilst catering to the different levels of digital capacity and capability.

### Finalisation

To ensure the **stability** and **finalisation** of the network, actors will develop standards, regulations and organisational norms to manage the use and distribution of *technologic innovations*. This process will normalise both the *sustainable* and *disruptive* elements of the *innovations*. For example, standard Web technologies have established an Open Web Platform, foundational for various data services from ingest to consumption (W3C, 2014, 2021). These technologies serve as cornerstones for Digital Humanities and cultural heritage pipelines, as well as guidelines such as the FAIR principles, aligning with software and tools used by *designated communities* (Lamprecht et al., 2020). Digital infrastructures must identify frameworks that position technologies and information processes (e.g. paradata), including *metadata crosswalks,* as active participants in the ecosystem and the *techno economic paradigm.* The *metadata crosswalk* and associated *technologic innovations* must ensure scalable, flexible data management and interoperability, facilitating access for subaltern and community groups. This is essential to prevent dominant institutions from overshadowing weaker actors when forming **triangles of interessement** in the network.

During this **finalisation** phase, it is crucial to create a unified narrative that engages the socio-economic and technical systems within the network. For the OCH ecosystem, this phase must rigorously test the scalability and flexibility of data management systems to ensure they support both community and subaltern groups, as well as large institutional or national organisations. Knowledge systems and infrastructures must be interoperable to address the needs of network members. They must also be flexible enough to integrate with other knowledge systems, worldviews, and technologies across different *techno economic paradigms*, ensuring long-term sustainability. Adopting approaches such as such as Infrastructures as a Service (IaaS), Platform as a Service (PaaS), and Data as a Service (DaaS) can greatly help the **finalising** or **stabilising** the OCH network. Perspectives such as IaaS can offer scalable, virtualised computing resources, helping organisations avoid the costs and complexities of maintaining physical servers. This allows infrastructures to adjust dynamically to changing demands. PaaS, in turn, will provide platforms that help simplify the development of applications and services, without the need to handle underlying infrastructures. DaaS delivers data in a readily consumable format, enabling non-expert users to generate value from *information objects* without engaging with complex systems. For the OCH ecosystem, *information objects* are critical. Thus, these infrastructures must ensure robust, adaptable digitisation and digitalisation processes, maintaining consistency across the sector’s complex knowledge. Systems must also balance copyright, access, and licensing challenges while implementing sharing models that respect intellectual property and data sovereignty. This balance can promote accessibility while safeguarding creator’s rights. Finally, actors will need to include an alignment of the *techno economic paradigm* that accounts for the engagement of stakeholders, training and guaranteeing effective use and adaptation of its infrastructures.

## Discussion

As we examine the core elements of the OCH ecosystem, it becomes evident that hierarchical and hegemonic structures often strengthen certain relationships while weakening others. CH organisations, bound by bureaucratic systems, frequently struggle to engage with communities, often focusing on internal agendas (Janes, 2013; Wilde & Mann, 2010). In response, some institutions are transitioning toward community-based models of collaboration (Roberts et al., 1997; Sinclair, 2003). However, to fully integrate these communities, a deeper understanding of the interactions and power dynamics is necessary (Falk & Dierking, 2012).

A key feature of the OCH ecosystem is its *convergence culture* (Jenkins, 2006), where multiple ecosystems begin cooperating between them, causing users to migrate to other systems. Many spaces outside the CH sector have become the primary hubs for cultural participation, knowledge engagement, and transmedia storytelling. These platforms are not only central to the production and consumption of *information objects*, but also to how users access and engage with CH content. Most Web searches related to CH content begin in Google or Wikipedia, or other external services rather than on the platforms of CH organisations that hold the original *information object* (Blackwood, 2014; Jansen & Spink, 2006; Harrop et al., 2015; Guldbæk Rasmussen et al., 2009). This shift has sparked ongoing debates about whether libraries and CH institutions should prioritise embedding access within external platforms rather than relying on their own discovery systems (Harrop et al., 2015).

Within digital collections, the relationship between the physical object and the institution that holds it can be overlooked by users. While platforms can ingest vast datasets, they may prioritise certain metadata elements over digital provenance, potentially obscuring the original context of the *information object*. In this sense, libraries and other GLAM institutions, play a crucial role in preserving digital materials, ensuring that digital research services engage with the full complexity of *information objects*, including born-digital data from *designated communities*.

The production of value or contextualisation of the knowledge embedded in *information objects* can be achieved through a variety of as-a-Service models, which address both infrastructural and knowledge needs across a wide range of actors. These models facilitate the sustainable management and use of digital collections, ensuring the integrity of *information objects* and their provenance remains central to knowledge production.

Analysing the **problematisation** phase can help identify challenges behind the production, management and engagement with *information objects* and their *designated communities*. Similar to open government data networks (Tinati et al., 2011), the CH sector does not consider end users as core actors in the network and solely as consumers of tools and software. This can become problematic, since *information objects* are the digital representations of the human and non-human knowledge of the world, which in turn, aid our understanding of how humans (and *designated communities)* interact with the world. It is through the socio-technical infrastructures and services offered in the OCH ecosystem, that the actors can produce value from it. In this sense, FAIR principles have served to safeguard the transparency, reusability, and reproducibility of data objects. However, it is important to highlight that FAIR principles should apply to all kinds of *information objects,* including algorithms, tools, workflows, pipelines, and software used to generate new knowledge, including scientific publications and exhibitions, among many others (Lamprecht et al., 2020). Furthermore, the process of identifying Designated Communities can be aided by FAIR principles. Nevertheless, in this process there is the need to consider that FAIR principles have been created from a starting point where different *designated communities* already have the same equity (not equality) which might require extending policies of data ethics to policies of data justice (D'ignazio and Klein, 2023).

The structure of the OCH ecosystem requires all actors of both, human and technical, to interact and be categorised equally. The OCH network is built from other networks, such as scholarly networks or scholarly social-machines, including citizen science platforms like Zooniverse, open access repositories like Zenodo, and other open science frameworks. Cultural heritage organisations can benefit by understanding how their system connect with other institutions, and platforms, avoiding isolation. During the **mobilisation** phase, cultural organisations must identify a wide range of actors to generate **enrolment** and establish sustainable models to preserve cultural datasets. In the UK, this includes the **enrolment** of funders like the AHRC or the National Lottery Heritage Fund and pinpointing their specific needs of **interessement.** OCH and CH actors need to recognise **systems of alliances** that might hinder or alienate other actors, leading to unbalanced, technologic disparities, and ineffective preservation of digital collections. Incurring in these disparities will make it more difficult for cultural heritage organisations to advocate for the necessary support from stakeholders such as government bodies and funders alike.

It might be difficult to make the most of other social-machines or networks within the OCH infrastructure whilst identifying the downsides or the specific **Obligatory Passage Point** that the network needs to engage with. Networks such as the OCH ecosystem can further extend the digital breach amongst the *designated communities*, where subaltern groups and their organisations can be excluded from participating in the OCH network and knowledge production. Actors in the OCH ecosystem must balance technical and human systems since overreliance on technology may lead to data and techno-centric perspectives that hinder ethical, cultural and data governance processes, which require human ‘intelligence’ and contextual understanding. That said, the **systems of alliances** in the OCH ecosystem call for a holistic understanding of how socio-technical systems could be used with an already established plan to identify technologic dependency and its obsolescence whilst providing the vision of human, organisational, economic and technical actors with real pragmatic strategies.

## Conclusion and further work

Approaching the OCH ecosystem through Callon’s **Process of Translation** offers a broader understanding of the complex connections between technology, human agency, and cultural narratives. The process of **problematisation** highlights key challenges for socio-technical systems, particularly within the OCH ecosystem, where technology plays a critical role. It transforms digital platforms into spaces for cultural and knowledge exchange, whilst becoming vital in preserving, disseminating, and enriching digital collections. However, as OCH operates as a social machine, it is vital to align stakeholders across the ecosystem through the process of **enrolment**. Although this primarily focuses on actors within the UK, it also considers broader issues of decolonisation, policy and data representation. These aspects are crucial when developing policy for the human and technical elements of the OCH ecosystem. Identifying this **mobilisation** phase through the **Process of Translation**, can aid cultural organisations in planning for *sustainable* and *disruptive technologies*, avoiding unintended consequences amongst their human actors or cultural datasets.

From a technical perspective, *disruptive technologies* are often difficult to identify until they have already affected digital infrastructures. Developing strategies that help organisations assess the broader impact(s) of both human and technical actors within their ecosystem can lead to sustainable models. These models must adapt to future technological innovations while preserving the integrity and authenticity of cultural assets, narratives and communities. Ensuring **stability** or **finalisation** involves keeping OCH and CH active and connected within the global network of human knowledge, both in digital and analogue space.

Human involvement is as crucial as the technology underpinning the OCH ecosystem. Effective collaboration among curators, academics, scholars, community leaders and policymakers is essential, though achieving a unified vision can be challenging. To enhance integration, it’s important to identify how actors and groups can build collaborative networks and systems of alliances. By embracing the OCH ecosystem, they can create structures, strategies, and approaches that strengthen partnerships and further their work with further social-machines that support their collaborative efforts.

This work enables a deeper deconstruction and engagement with each stage of the **Process of Translation**, ensuring every phase is approached with sensitivity, foresight, and commitment to a global set of knowledge and worldviews. It involves embracing technological advancements that enrich and support cultural knowledge. By exploring the OCH ecosystem through a socio-technical deconstruction, we lay the groundwork for a future where cultural heritage is preserved and actively re-used, celebrated and reimagined in the digital world. This emphasises the need for OCH to evolve as an inclusive, dynamic and responsible platform. The OCH ecosystem should foster true integration and harmonisation of technology, culture and diverse human interactions. Deconstructing digital infrastructures like OCH through the **Process of Translation** lens can build a long-term, sustainable ecosystem for the CH sector, ensuring the preservation, interpretation, and sharing of cultural narratives in a way that engages and resonates with a global audience.

## Data Availability

Data presented in Fig. 3 was produced through Europeana’s SPARQL Endpoint by retrieving each institution and the count of resources they provide. Find resources and their associated data providers and group the results by institution.

## Footnotes

1 E-infrastructure includes computers and software for modelling, simulation, and data analysis; data storage, curation, and archiving; software platforms for managing data, security, and workloads; networks enabling data access and transfer; and experts in scientific software and data-intensive science to maintain and develop these interconnected systems (STFC UKRI, 2017).

2 FAIR – Findable, Accessible, Interoperable, Reusable (Wilkinson et al., 2016).

3 CARE – Collective benefit, Authority control, Responsibility and Ethics (Carroll et al., 2020).

4 FLOK stands for "Free/Libre Open Knowledge," promoting free and open use of knowledge. It aims to establish and use this shared knowledge for community well-being as a societal goal. FLOK seeks to offer alternatives that challenge destructive behaviours by presenting possible solutions for the betterment of humanity.

5 https://www.nationalcollection.org.uk/

6 https://www.ukri.org/what-we-do/browse-our-areas-of-investment-and-support/ukri-world-class-laboratories-fund/

7 https://www.ukri.org/opportunity/ahrc-capability-for-collections-fund/

8 https://www.ukri.org/opportunity/renew-and-upgrade-facilities-for-creative-and-cultural-research/

9 Department for Business, Energy and Industrial Strategy.

10 Department for Levelling Up, Housing and Communities.

11 Department for Digital, Culture, Media and Sport.

## Glossary

**Application profile**: “…a metadata design specification that uses a selection of terms from multiple metadata vocabularies, with added constraints, to meet application-specific requirements”. (DCMI, 2024)
**CARE principles**: Principles for Indigenous Data Governance: Collective benefit, Authority control, Responsibility and Ethics.
**Convergence culture**: Term coined by Jenkins (2006) to explain the flow of content and media across multiple digital platforms, the cooperation between industries, and new behaviours of audiences who will explore further for the necessary digital experiences they are looking for.
**Data objects / Information object**: The coupling of objects, metadata, information, and digital surrogates are no longer concepts that exist in isolation and require to be contextualized and adapted for the diverse sectors, disciplines, and domains from the heritage and cultural sector and SIC areas. This coupling of object-process has been defined by The Open Archive Information System (OAIS) as an information object.
**Designated community**: The wide range of potential and theoretical users or audiences of any given information object.
**Disruptive innovation**: Type of digital innovation that takes place through ground-breaking technologies that produce new organisational paradigms and can enable new products or services to emerge, whilst pushing users or audiences to re-adapt.
**Enrolment**: For innovation to take place, networks or social groups such as research communities, research councils, and policymakers must engage in the process of enrolment.
**FLOK / FLOK society**: FLOK stands for "Free/Libre Open Knowledge," which means promoting the use and sharing of knowledge freely and openly. It aims to establish and use this shared knowledge in a country that values community well-being as a societal goal. FLOK seeks to offer alternatives that challenge destructive behaviours by presenting possible solutions for the betterment of humanity.
**Interessement**: Process where the focal actor aligns the interests of other actors through negotiation and strategic devices to secure their participation and commitment to the network or ecosystem.
**Knowledge infrastructure/systems**: Knowledge Infrastructures exist in the process of translation through scholarly social-machines which focus on the inclusive and collaborative knowledge generation ecosystem and thus help overcome customary barriers to scholarly activity.
**Metadata crosswalk**: A process in which cultural heritage organisations create and implement policies and guidelines to ensure interoperability and standardisation across different metadata schemas, making use of terms from multiple metadata vocabularies and frameworks.
**Mobilisation**: The process where actors adapt and refine innovations in response to feedback, aligning all participants to stabilise or finalise the network.
**Paradata**: Data that documents the processes and contexts in which digital assets are created (Bentkowska-Kafel & Denard, 2012).
**Problematisation**: Process actors identify and frame a problem that needs to be addressed, their role within the network’s structure and their roles around the solution of that problem.
**Process of translation**: A core concept in Actor-Network Theory that examines how human and non-human actors form networks through the sequential stages of problematisation, interessement, enrolment, and mobilisation, ultimately stabilising and transforming collective actions and relationships.
**Sustaining innovation**: Sustaining innovations can help extend and reinforce existing services and products while improving performance or affecting how they cater for specific user needs.
**Stabilisation/finalisation**: The process where the network solidifies through the establishment of standards, regulations, and norms, ensuring the sustained and consistent use and distribution of innovations and practices among all actors.
**System of alliances**: A network of interconnected and cooperative relationships among actors, formed to support and sustain collective goals.
**Techno-economic paradigm**: The interdependent relationship between technological innovations and the socio-economic structures within which they operate.
**Technologic trajectory**: The path and rate of technological development and innovation within a specific field. It is influenced by both scientific advancements and socio-economic factors, which guide the direction and focus of future technological efforts.
**Triangle of interessement**: A more focused **system of alliances** that involves a subset of key actors whose aligned interests form a strong and stable core, crucial for the network's initial stability and strength.
